# Oculocerebrorenal syndrome of Lowe (OCRL) controls leukemic T-cell survival by preventing excessive PI(4,5)P_2_ hydrolysis in the plasma membrane

**DOI:** 10.1016/j.jbc.2023.104812

**Published:** 2023-05-11

**Authors:** Huanzhao Chen, Chen Lu, Yuhui Tan, Marion Weber-Boyvat, Jie Zheng, Mengyang Xu, Jie Xiao, Shuang Liu, Zhiquan Tang, Chaofeng Lai, Mingchuan Li, Vesa M. Olkkonen, Daoguang Yan, Wenbin Zhong

**Affiliations:** 1 MOE Key Laboratory of Tumor Molecular Biology, Jinan University, Guangzhou, China; 2 Minerva Foundation Institute for Medical Research, Biomedicum 2U, Helsinki, Finland; 3 Department of Anatomy, Faculty of Medicine, University of Helsinki, Helsinki, Finland; 4 Charité Universitätsmedizin Berlin, Corporate Member of Freie Universität Berlin and Humboldt-Universität zu Berlin, Institute of Neurophysiology, Berlin, Germany; 5 Affiliated Cancer Hospital & Institute of Guangzhou Medical University, Guangzhou, China

**Keywords:** Ca^2+^ homeostasis, oculocerebrorenal syndrome of Lowe 1, OSBP-related protein 4L, phosphatidylinositol 4,5-bisphosphate, T-cell acute lymphoblastic leukemia

## Abstract

T-cell acute lymphoblastic leukemia (T-ALL) is one of the deadliest and most aggressive hematological malignancies, but its pathological mechanism in controlling cell survival is not fully understood. Oculocerebrorenal syndrome of Lowe is a rare X-linked recessive disorder characterized by cataracts, intellectual disability, and proteinuria. This disease has been shown to be caused by mutation of oculocerebrorenal syndrome of Lowe 1 (*OCRL1*; OCRL), encoding a phosphatidylinositol 4,5-bisphosphate [PI(4,5)P_2_] 5-phosphatase involved in regulating membrane trafficking; however, its function in cancer cells is unclear. Here, we uncovered that *OCRL1* is overexpressed in T-ALL cells, and knockdown of *OCRL1* results in cell death, indicating the essential role of OCRL in controlling T-ALL cell survival. We show OCRL is primarily localized in the Golgi and can translocate to plasma membrane (PM) upon ligand stimulation. We found OCRL interacts with oxysterol-binding protein–related protein 4L, which facilitates OCRL translocation from the Golgi to the PM upon cluster of differentiation 3 stimulation. Thus, OCRL represses the activity of oxysterol-binding protein–related protein 4L to prevent excessive PI(4,5)P_2_ hydrolysis by phosphoinositide phospholipase C β3 and uncontrolled Ca^2+^ release from the endoplasmic reticulum. We propose *OCRL1* deletion leads to accumulation of PI(4,5)P_2_ in the PM, disrupting the normal Ca^2+^ oscillation pattern in the cytosol and leading to mitochondrial Ca^2+^ overloading, ultimately causing T-ALL cell mitochondrial dysfunction and cell death. These results highlight a critical role for OCRL in maintaining moderate PI(4,5)P_2_ availability in T-ALL cells. Our findings also raise the possibility of targeting *OCRL1* to treat T-ALL disease.

The gene responsible for Lowe syndrome was identified by positional cloning of X chromosome breakpoints, designated oculocerebrorenal syndrome of Lowe 1 (*OCRL1)* (1), and the mutations of this gene are associated with both the oculocerebrorenal syndrome of Lowe and Dent disease (2). OCRL was originally described as a Golgi-localized protein in fibroblasts and epithelial cells (3). Subsequent reports suggested that OCRL is localized to the Golgi (4, 5), PM (6), early endosomes (7, 8), lysosomes (9), and clathrin-coated trafficking intermediates (4, 10). OCRL can also be detected in the PM when quiescent cells are stimulated with growth factors, suggesting that it translocates to the PM. This may be mediated by Rac1, a member of the Rho GTPase family, which forms a stable complex with the C-terminal Rho GAP-like domain of OCRL (5, 6). Furthermore, there are two clathrin-binding sites and a clathrin adaptor AP-2 binding site present in OCRL (11), which play important roles in clathrin-mediated endocytosis defects (12). The interaction of OCRL with clathrin regulates protein trafficking between the Golgi and endosomes (4).

Human inositol 5-phosphatases form a family of 10 members, and their catalytic substrates include inositol 1,4,5-trisphosphate (IP_3_), inositol 1,3,4,5-tetrakisphosphate, phosphatidylinositol 3,4,5-trisphosphate, and phosphatidylinositol 4,5-bisphosphate [PI(4,5)P_2_] (13). OCRL has a marked preference for PI(4,5)P_2_ compared with the other inositol 5-phosphatases, suggesting that it is mainly a PI(4,5)P_2_ 5-phosphatase, that produces phosphatidylinositol 4-phosphate [PI(4)P] (14). In addition, PI(4,5)P_2_ is a precursor of IP_3_ and diacylglycerol after phosphoinositide phospholipase C (PLC) activation, which is essential for early signaling of cell surface receptors. Among them, IP_3_ from PLC-mediated inositol phosphate breakdown induces Ca^2+^ release from the endoplasmic reticulum (ER) (15). PLC-mediated activation of PI(4,5)P_2_ hydrolysis and the Ca^2+^ is a key intracellular messenger that controls different cellular functions but causes cell death when the homeostasis is broken (16). Ca^2+^ released from the ER is taken up by mitochondria through the mitochondrial Ca^2+^ uniporter (MCU) (17). Furthermore, stimulation of mitochondrial oxidative metabolism by Ca^2+^ is generally considered important for the control of cellular ATP homeostasis (18). Excessive Ca^2+^ taken up by MCU into mitochondria results in mitochondrial Ca^2+^ overload, disrupting mitochondrial permeability transition pore opening, release of cytochrome c and activation of caspase3/7 (19). Thus, the content of PI(4,5)P_2_ in the PM used for PLC hydrolysis should be controlled to maintain Ca^2+^ homeostasis.

Oxysterol-binding protein (OSBP)-related protein 4 (ORP4; also known as OSBP2) is a member of the oxysterol-binding protein–related protein (ORP) family, which are described as lipid-binding/transfer proteins with variable tissue expression, ligand specificity, and subcellular localization and function (20). It has three recognized variants, OSBP-related protein 4L (ORP4L), ORP4M, and ORP4S. All together 23 exons are required to encode the full-length ORP4L protein, including the OSBP-related ligand-binding domain, pleckstrin homology domain, and a motif designated two phenylalanines in an acidic tract, to facilitate lipid transfer and signaling activities (21). Our previous study showed that ORP4L promotes phospholipase C β3 (PLCβ3) translocation from the nucleus to the PM (22) and forms a PLCβ3-Gαq/11-CD3ε complex that activates PLCβ3 upon anti-cluster of differentiation 3 (CD3) stimulation, regulating IP_3_ production and ER Ca^2+^ release in T-ALL cells (23). ORP4L extracts PI(4,5)P_2_ from the PM and presents it to PLCβ3, enabling IP_3_ generation, and subsequent Ca^2+^-dependent bioenergetics and cell survival (24).

The CD3 molecule is an important marker of the surface of T cells, consisting of five polypeptide chains of γ, δ, ε, ζ, and η, which are involved in the assembly, stabilization and signal transduction of the TCR–CD3 complex (25). Anti-human CD3 monoclonal antibodies recognize ε chains of CD3 molecules on the TCR-CD3 complex, thereby enhancing the activation and proliferation of T lymphocytes (26). In normal T cells, upon stimulation with anti-CD3, LAT (pp36) is heavily tyrosine phosphorylated and subsequently binds to PLCγ1 to increase IP_3_ and intracellular Ca^2+^ in a G protein–independent manner (27). This process is required for the recruitment of the CD3ζ chain to active ZAP70, which plays a central role in CD3 signaling (28). However, CD3ζ chain expression is defective in T-ALL cells (29). Thus, CD3 signaling is swapped to Gαq/11 in the presence of ORP4L and sequentially activates Gαq/11 and PLCβ3, which becomes the dominant enzyme for IP_3_ generation and intracellular Ca^2+^ homeostasis in T-ALL cells (23).

In this study, we show that OCRL directly binds to ORP4L which stimulates OCRL translocation from the Golgi to the PM, maintaining moderate PI(4,5)P_2_ consumption by PLCβ3 and Ca^2+^ homeostasis for T-ALL cell survival.

## Results

### Elevated *OCRL1* expression is essential for T-ALL cell survival

**Figure 1:**
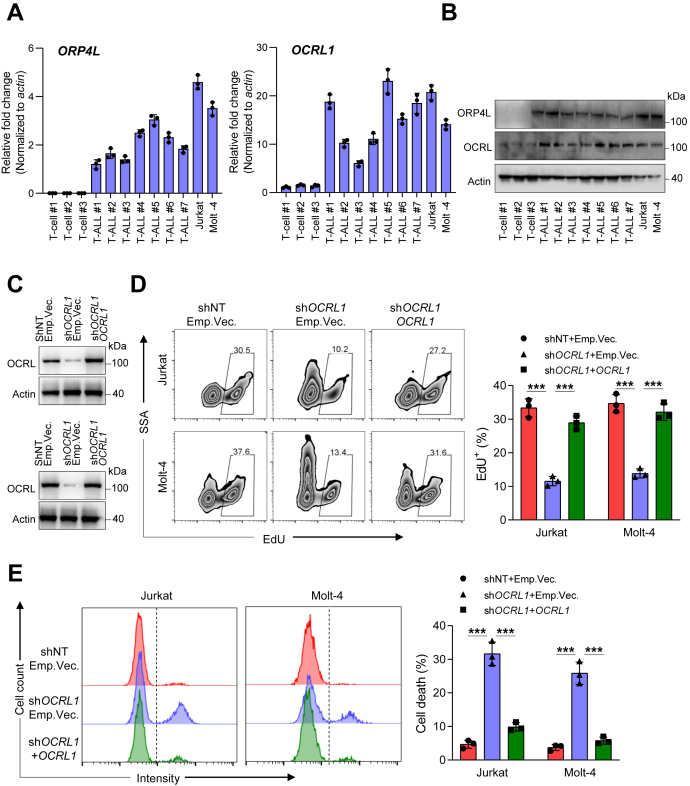
**Elevated *OCRL1* expression in T-ALL cells maintains cell survival.***A*, qRT-PCR analysis of *ORP4L* (left) and *OCRL1* (right) mRNA expression in normal human T-cells, primary T-ALL cells, and two T-ALL cell lines, Jurkat T-cells and Molt-4 cells. The mRNA expression level is calculated relative to *actin* expression. Values are shown as the mean ± S.D. of three repeats folded over the T-ALL #1 group (for *ORP4L*) or T-cell #1 group (for *OCRL1*), respectively. *B*, Western blot analysis of ORP4L and OCRL protein expression in normal human T-cells, primary T-ALL cells, and two T-ALL cell lines. *C*, Western blot showing OCRL expression in Jurkat T-cells (*upper*) and Molt-4 cells (*lower*) subjected to control (shNT), *OCRL1* knockdown (sh*OCRL1*) alone, or combined with *OCRL1* rescued expression (sh*OCRL1*+*OCRL1*). Cells were transduced with lentivirus expressing control nontargeting shRNA or *OCRL1*-specific shRNA for 48 h before transduction with lentivirus expressing *OCRL1*. After additional 48 h of culture, the cells were used for analysis. *D*, cell proliferation analysis of Jurkat T-cells and Molt-4 cells subjected as in panel *C*. Cells were incubated with EdU for 1 h and analyzed by flow cytometer. *E*, cell death analysis of Jurkat T-cells and Molt-4 cells subjected as in panel *C*. Cells were stained by LIVE/DEAD Fixable Dead Cell Stain Kits and analyzed by flow cytometer. The data represent mean ± SD (n = 3). ∗∗∗*p* < 0.001; Student's *t* test. EdU, ethynyl-deoxyuridine; OCRL, oculocerebrorenal syndrome of Lowe 1; ORP4L; OSBP-related protein 4L; OSBP, oxysterol-binding protein; T-ALL, T-cell acute lymphoblastic leukemia.

As OCRL catalyzes hydrolysis of PI(4,5)P_2_, the same substrate that PLCβ3 uses, we explored the relationship between OCRL and ORP4L. As we previously reported (23), *ORP4L* mRNA and protein are undetectable in normal T-cells, but they are upregulated in primary T-ALL cells and two T-ALL cell lines, Jurkat T-cells and Molt-4 cells (Fig. 1, *A* and *B*). *OCRL1* is expressed in normal T-cells, but it is significantly elevated in T-ALL cells (Fig. 1, *A* and *B*). By analyzing 1036 human cancer cell lines in CCLE database (https://portals.broadinstitute.org/ccle), we found *OCRL1* was expressed in T-ALL cell lines (Fig. S1*A*). To further determine *OCRL1* expression in T-ALL cells, the assessment of human leukemia databases (GSE48558) showed a significant increase in *OCRL1* expression in patients’ T-ALL cells and T-ALL cell lines compared with that in normal T-cells (Fig. S1*B*). Thus, we identified upregulation of *OCRL1* in T-ALL cells.

To investigated the role of OCRL in T-ALL cell survival, we transduced Jurkat and Molt-4 cells with lentivirus carrying sh*OCRL1* alone or combined with *OCRL1* cDNA to rescue its expression. *OCRL1* expression was significantly knockdown by shRNA but can rescue after cotransduction of *OCRL1* cDNA, which were confirmed by Western blot (Fig. 1*C*). By performing cell proliferation experiments, we found that cells with knockdown of *OCRL1* display a reduced percentage of cells with ethynyl-deoxyuridine (EdU) in newly synthesized DNA, indicating decreased cell proliferation (Fig. 1*D*). Simultaneously, cell death analysis revealed that knockdown of *OCRL1* increased cell death (Fig. 1*E*), whereas, these phenotypes were abolished in *OCRL1* expression rescued cells (Fig. 1, *D* and *E*). To avoid the off-target effect of shRNA, we confirmed the effects of *OCRL1* knockdown by using an independent shRNA (Fig. S1, *C*–*E*). Take together, we evidenced that elevated *OCRL1* expression is essential for T-ALL cell survival.

### ORP4L is a novel binding partner of OCRL in T-ALL cells

**Figure 2:**
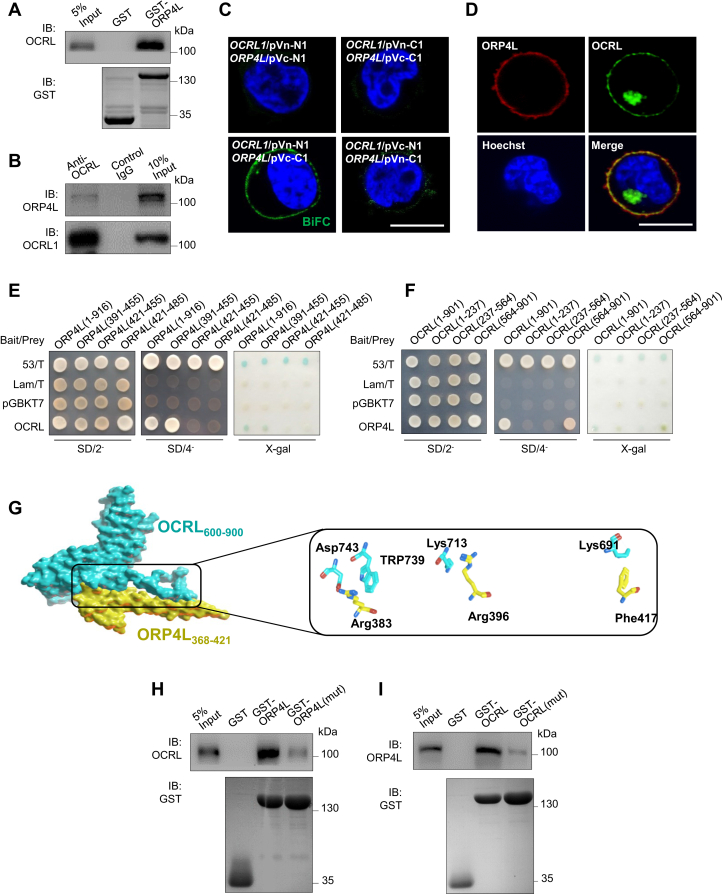
**The interaction of OCRL and ORP4L.***A*, OCRL interacts with ORP4L in GST-pulldown assays. Lysates derived from Jurkat T-cells were incubated with glutathione-agarose beads preloaded with the GST or GST-ORP4L fusions. The retained proteins were eluted, fractionated by 10% SDS/PAGE, and immunoblotted with anti-OCRL antibody. Approximately equal amounts of different GST fusions were immobilized on glutathione-agarose beads in these assays, as verified by immunoblotted with anti-GST antibody. *B*, coimmunoprecipitation of endogenous OCRL and ORP4L in Jurkat T-cells; precipitation was carried out with 5 μg OCRL antibody or control-IgG followed by Western blot analysis with antibodies against ORP4L or OCRL. *C*, visualization of interaction among OCRL and ORP4L in living cells using BiFC analysis. Fluorescence images of Jurkat T-cells transfected with plasmid encoding *OCRL1* or *ORP4L* fused to the Venus protein fragments as indicated were acquired 36 h after transfection. Scale bar, 10 μm. *D*, colocalization of ORP4L (*red*) and OCRL (*green*) in plasma membrane of Jurkat T-cells. Nucleus, *blue*. Scale bar, 10 μm. *E*, the full-length *OCRL1* in bait vector pGBKT7 and truncated fragments of *ORP4L* in prey vector pGADT7 cotransformed into the yeast were grown on SD/2-; 53/T is a positive control, and Lam/T is a negative control; the interaction was monitored by growth on SD/4- and the use of X-gal assay. *F*, the full-length *ORP4L* in bait vector pGBKT7 and truncated fragments of *OCRL1* in prey vector pGADT7 cotransformed into the yeast were grown on SD/2-; 53/T is a positive control, and Lam/T is a negative control; the interaction was monitored by growth on SD/4- and the use of X-gal assay. *G*, predicted binding mode of ORP4L (*yellow*) to OCRL (*blue*) and the site view show the detailed maps of key interaction residues of ORP4L (*yellow*) and OCRL (*blue*) complex. *H* and *I*, OCRL interacts with ORP4L and ORP4L(mut) (*H*), or ORP4L interacts with OCRL and OCRL (mut) (*I*) in GST-pulldown assays. Lysates derived from Jurkat T-cells were incubated with glutathione-agarose beads preloaded with the indicated GST fusions. The retained proteins were eluted, fractionated by 10% SDS/PAGE, and immunoblotted with anti-OCRL antibody (*H*) or anti-ORP4L (*I*). Approximately equal amounts of different GST fusions were immobilized on glutathione-agarose beads in these assays, as verified by immunoblotted with anti-GST antibody. Bimolecular fluorescence complementation. BiFC, bimolecular fluorescence complementation; GST, glutathione S-transferase; OCRL, oculocerebrorenal syndrome of Lowe 1; ORP4L; OSBP-related protein 4L; OSBP, oxysterol-binding protein.

ORP4L was important for T-ALL cell Ca^2+^ homeostasis and cell survival (23). As both OCRL and ORP4L regulate PM PI(4,5)P_2_ and require for T-ALL cell survival, we further study their relationship in Jurkat T-cells. We first tested whether they interact with each other by a series of experiments. In a pull-down assay using lysates from *OCRL1*-overexpressing Jurkat T-cells, the OCRL protein was pulled down by glutathione S-transferase (GST)-ORP4L but not by GST (Fig. 2*A*). We carried out immunoprecipitation experiments in Jurkat T-cells and detected an interaction between endogenous OCRL and ORP4L proteins (Fig. 2*B*). Bimolecular fluorescence complementation (BiFC) provides the potential for direct visualization of protein interactions in living cells (30). To investigate the interaction between OCRL and ORP4L in living cells, expression vectors encoding the Venus fusion protein fragments were cotransfected into Jurkat T-cells. After 36 h, heterodimer fluorescence was observed at the PM of the cells transfected with *OCRL1*/pVn-N1 and *ORP4L*/pVc-C1 (Fig. 2*C*). Furthermore, confocal fluorescence microscopy analysis revealed a largely Golgi distribution of OCRL, but some of OCRL also localized at the PM, overlapping with the ORP4L staining in Jurkat T-cells (Fig. 2*D*). These results evidenced that OCRL interacts with ORP4L in T-ALL cells.

In order to map which region of ORP4L is involved in the interaction with OCRL, deletion constructs of *ORP4L* were inserted in the prey plasmid pGADT7, and their interactions with OCRL were evaluated by using the yeast 2-hybrid assay (Fig. 2*E*). The positive control 53/T and negative control Lam/T demonstrated the reliability of these experiments. None of the deletion constructs of *ORP4L* grew on SD/4- plates or activated the X-gal reporter gene when combined with the empty plasmid pGBKT7, indicating that ORP4L did not autoactivate the reporter. The prey constructs containing full-length *ORP4L* or the segment containing aa 391 to 455 could grow on SD/4- plates and activated the X-gal reporter, indicating that this region in ORP4L interacts with OCRL. Conversely, to identify the region of OCRL that is sufficient for interaction with ORP4L, truncated fragments of *OCRL1* were inserted in the prey plasmid pGADT7. When combined with the empty plasmid pGBKT7, none of the deletion constructs of *OCRL1* grew on SD/4- plates or activated the X-gal reporter. The prey constructs containing full-length *OCRL1* or the segment containing aa 564 to 901 grew on SD/4- plates and activated the X-gal reporter, suggesting that aa 564 to 901 of OCRL are sufficient for the interaction with ORP4L (Fig. 2*F*).

To further determine the key amino acids mediating the binding between OCRL and ORP4L, we predicted the binding mode of ORP4L to OCRL by molecular docking. Asp743/Trp739 of OCRL are in contact with Arg383 of ORP4L, and Lys713 of OCRL is in contact with Arg396 of ORP4L. Furthermore, Lys991 of OCRL is in contact with Phe417 of ORP4L (Fig. 2*G*). We further mutated the predicted binding site in ORP4L (the Arg383, Arg396, Phe417) and OCRL (Asp743, Trp739, Lys713, Lys691). In the pull-down assay, as compared to wildtype ORP4L, the OCRL protein pulled down by GST-ORP4L (mut) was dramatically reduced (Fig. 2*H*), and the ORP4L protein pulled down by GST-OCRL (mut) was also decreased (Fig. 2*I*), indicating the requirement of the Arg383, Arg396, and Phe417 site of ORP4L and the Asp743, Trp739, Lys713, and Lys691 site of OCRL for their interaction.

In order to use this OCRL (mut) for functional study, we next detected its localization and phosphatase activity. For these purposes, we overexpressed Xpress-tagged *OCRL1* or *OCRL1*(mut) in Jurkat T-cells, then isolated the PM and Golgi fractionations to analyze their localization. We verified PM and Golgi purification by the corresponding marker proteins (Fig. S2*A*). We found the equal amount of OCRL and OCRL (mut) protein in PM and Golgi (Fig. S2*B*). Immunofluorescence staining provided further evidence that OCRL (mut) did not alter its intracellular localization (Fig. S2*C*). For *in vitro* phosphatase activity, we purified recombinant OCRL and OCRL (mut) proteins and incubated them with PI(4,5)P_2_ containing PM and then measured the conversion of PI(4,5)P_2_ to PI(4)P. Both the OCRL and OCRL (mut) increased the PI(4)P production and reduced the PI(4,5)P_2_ levels (Fig. S2*D*) with the similar activity, indicating that this *OCRL1* mutation did not affect its phosphatase activity.

### OCRL is translocated from the Golgi to the PM upon anti-CD3 stimulation in ORP4L-dependent manner

It has been found that after Rac activation, OCRL is translocated to the PM and accumulates specifically in membrane ruffles (6). As a protein that directly interacts with OCRL, ORP4L has been shown to have the ability of assisting in protein translocation to the PM (23). So, we explored whether OCRL can translocate to the PM in T-ALL cells and whether ORP4L plays a role in this process.

**Figure 3:**
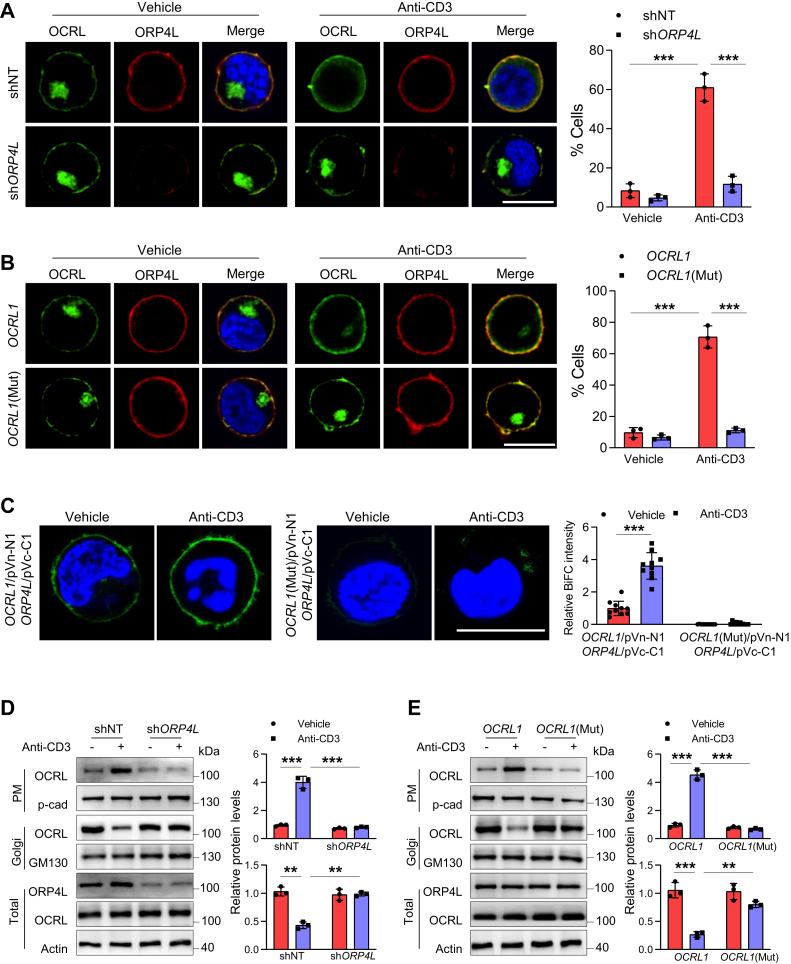
**Analysis of OCRL translocation in Jurkat T-cells upon anti-CD3 stimulation.***A*, confocal microscopy analysis of endogenous OCRL (*green*) and ORP4L (*red*) localization in shNT- or sh*ORP4L*-transfected Jurkat T-cells. Cells were stimulated with or without anti-CD3 (10 μg ml^−1^) for 5 min. Scale bars, 10 μm. The histograms represent the percentage of cells with OCRL translocation predominant PM localization. The percentage of cells that represent OCRL distribution upon anti-CD3 stimulation was quantified from >50 cells of each group. The histograms showed the results from three independent experiments. *B*, cells with wildtype *OCRL1* or *OCRL1* (mut) overexpression, confocal microscopy analysis of OCRL (by Xpress) and ORP4L in Jurkat T-cells after 5-min anti-CD3 stimulation (10 μg ml^−1^). Scale bars, 10 μm. The histograms represent the percentage of cells with OCRL translocation predominant PM localization. The percentage of cells that represent OCRL1 distribution upon anti-CD3 stimulation was quantified from >50 cells of each group. The histograms showed the results from three independent experiments. *C*, BiFC analysis between *OCRL1*/pVn-N1 and *ORP4L*/pVc-C1 or *OCRL1* (mut)/pVn-N1 and *ORP4L*/pVc-C1 in Jurkat T-cells. After transfected with the fragments for 24 h, cells were stimulated with or without anti-CD3 (10 μg ml^−1^) for 5 min and analyzed. Scale bars, 10 μm. The histograms showed the relative BiFC intensity from 10 cells. *D*, OCRL protein levels in PM, Golgi, and total lysate of Jurkat T-cells with ORP4L knockdown. Cells were stimulated with 10 μg ml^−1^ anti-CD3 for 5 min. The histograms showed the relative OCRL protein levels in PM (*upper*) and Golgi (*lower*) from three independent experiments. *E*, OCRL protein levels in PM, Golgi, and total lysate of Jurkat T-cells with *OCRL1* or *OCRL1* (mut) overexpression. Cells were stimulated with 10 μg ml^−1^ anti-CD3 for 5 min. OCRL were detected by Xpress antibody. The histograms showed the relative OCRL protein levels in PM (upper) and Golgi (lower) from three independent experiments. ∗∗*p* < 0.01; ∗∗∗*p* < 0.001; Student's *t* test. BiFC, bimolecular fluorescence complementation; CD3, cluster of differentiation 3; OCRL, oculocerebrorenal syndrome of Lowe 1; OSBP; oxysterol-binding protein, ORP4L; OSBP-related protein 4L; PI(4,5)P_2_, phosphatidylinositol 4,5-diphosphate; PM, plasma membrane.

Confocal imaging analyses showed that endogenous OCRL clustered in Golgi of unstimulated Jurkat T-cells, with a small amount of OCRL distribution at the PM where it colocalized with ORP4L. However, anti-CD3 stimulation induced a striking OCRL translocation from the Golgi to the PM and increased its colocalization with ORP4L (Fig. 3*A*). In contrast, translocation of OCRL was not observed in ORP4L-depleted cells, and the percentage of cells that responds to anti-CD3 stimulation was significantly reduced (Fig. 3*A*).

Next, we examined the translocation of exogenous overexpressed Xpress-tagged OCRL upon anti-CD3 stimulation in Jurkat T-cells. The results showed that anti-CD3 stimulation similarly induced exogenous OCRL translocation from the Golgi to the PM and increased its colocalization with ORP4L (Fig. 3*B*). However, this translocation was not observed for the OCRL (mut) incapable of binding to ORP4L (Fig. 3*B*). In BiFC assay, the OCRL and ORP4L can evoked a BiFC signal, which was enhanced upon anti-CD3 stimulation; however, these signals were undetectable in OCRL (mut) and ORP4L-transfected cells (Fig. 3*C*). Cell fractionation followed by the Western blot analysis provided further evidence for the translocation of OCRL from the Golgi to the PM upon anti-CD3 stimulation (Fig. 3, *D* and *E*). Anti-CD3 stimulation increased the OCRL protein level in PM, decreased it in Golgi, but these phenotypes were abolished in ORP4L knockdown cells (Fig. 3*E*). Similarly, anti-CD3 stimulation results in increase of exogenous OCRL, but not OCRL (mut) protein translocation from Golgi to PM (Fig. 3*E*). These results suggested that with the assistance of ORP4L, anti-CD3 stimulation activated OCRL translocation from the Golgi to the PM.

### OCRL mediates PI(4,5)P_2_ consumption at the PM by binding ORP4L

PI(4,5)P_2_ is a major inositol 5-phosphatase substrate that shows signaling properties, and its content in membranes could be affected by PI phosphatase activities (13). Since ORP4L-mediated PLCβ3 translocation also triggered the hydrolysis of PI(4,5)P_2_, we further analyzed the changes of PI(4,5)P_2_ after OCRL translocation to the PM in Jurkat T-cells.

**Figure 4:**
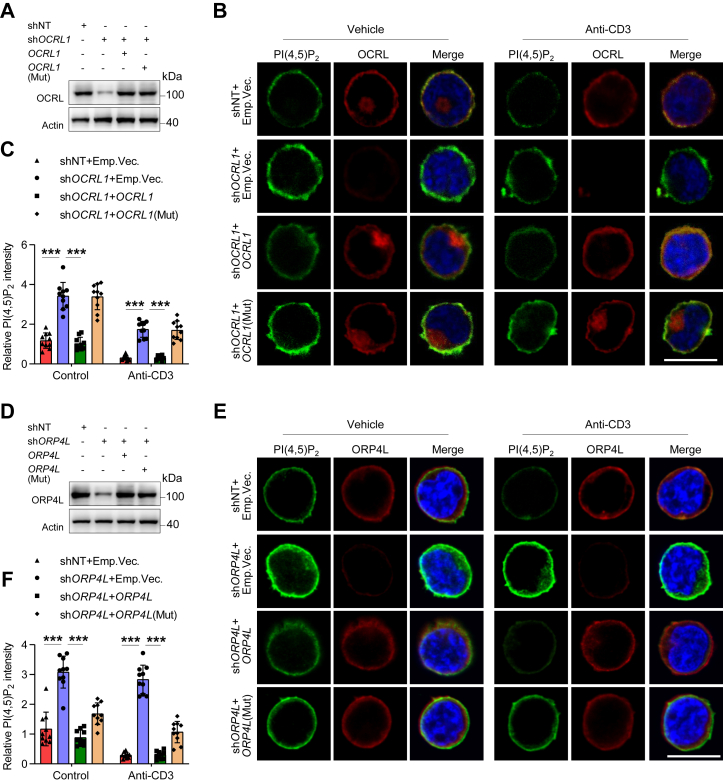
**OCRL-ORP4L interaction is required for PI(4,5)P**_**2**_**consumption in the PM.***A*, Western blot showing OCRL expression in Jurkat T-cells expressing PI(4,5)P_2_ probe GFP-PH_PLCδ1_. Cells were subjected to control (shNT), *OCRL1* knockdown (sh*OCRL1*) alone, or combined with *OCRL1* rescued expression (sh*OCRL1*+*OCRL1*) or combined with *OCRL1*(Mut) rescued expression (sh*OCRL1*+*OCRL1*(Mut)). Cells were transduced with lentivirus expressing shNT or sh*OCRL1* for 48 h, before transduction with lentivirus expressing *OCRL1* or *OCRL1*(Mut). After additional 48 h of culture, the cells were used for analysis. *B*, PI(4,5)P_2_ levels at the PM as detected by PI(4,5)P_2_ probe GFP-PH_PLCδ1_ in Jurkat T-cells treated as panel (*A*). Cells were stimulated with 10 μg ml^−1^ anti-CD3 for 5 min before analyzed. The cells with *OCRL1* knockdown or rescue expression were considered for analysis, and the relative PI(4,5)P_2_ levels expressed as fold change are shown in *C*. The data represent mean ± SD (n = 10 cells). Scale bar, 10 μm. *D*, Western blot showing ORP4L expression in Jurkat T-cells expressing PI(4,5)P_2_ probe GFP-PH_PLCδ1_. The cells subjected to control (shNT), *ORP4L* knockdown (sh*ORP4L*) alone, or combined with *ORP4L* rescued expression (sh*ORP4L*+*ORP4L*) or combined with *ORP4L* (Mut) rescued expression (sh*ORP4L* + *ORP4L* (Mut)). Cells were transduced with lentivirus expressing shNT or sh*ORP4L* for 48 h, before transduction with lentivirus expressing *ORP4L* or *ORP4L* (Mut). After additional 48 h of culture, the cells were used for analysis. *E*, PI(4,5)P_2_ levels at the PM as detected by PI(4,5)P_2_ probe GFP-PH_PLCδ1_ in Jurkat T-cells treated as panel (*D*). Cells were stimulated with 10 μg ml^−1^ anti-CD3 for 5 min before analyzed. The cells with *ORP4L* knockdown or rescue expression were considered for analysis, and the relative PI(4,5)P_2_ levels expressed as fold change are shown in *F*. The data represent mean ± SD (n = 10 cells). Scale bar, 10 μm. ∗∗∗*p* < 0.001; Student's *t* test. CD3, cluster of differentiation 3; OCRL, oculocerebrorenal syndrome of Lowe 1, OSBP, oxysterol-binding protein; ORP4L, OSBP-related protein 4L; PI(4,5)P_2_, phosphatidylinositol 4,5-diphosphate; PLC; phosphoinositide phospholipase C; PM, plasma membrane.

In Jurkat T-cells with *OCRL1* knockdown, wildtype *OCRL1* or *OCRL1* (mut) were re-overexpressed for rescue experiments (Fig. 4*A*). We analyzed the PI(4,5)P_2_ contents in PM by using PI(4,5)P_2_ indicator GFP-PH_PLCδ1_ domain (31). In the resting cells, *OCRL1* knockdown resulted in PI(4,5)P_2_ abundantly accumulated at the PM. Anti-CD3 stimulation decreased the PI(4,5)P_2_ level in control cells, but this decrease were reduced in *OCRL1* knockdown cells (Fig. 4, *B* and *C*). In rescue experiments, *OCRL1* re-overexpression abolish the effects on PI(4,5)_2_ accumulation, whereas no rescue was observed with *OCRL1*(mut) (Fig. 4, *B* and *C*). As a second method, the PMs were isolated for dot-blot to detect PI(4,5)P_2_ contents. We found the similar results as analysis by PI(4,5)P_2_ indicator (Fig. S3*A*).This observation indicated that OCRL consumed PI(4,5)P_2_ at the PM dependent on its binding to ORP4L.

Similarly, in Jurkat T-cells with *ORP4L* knockdown, wildtype *ORP4L* or *ORP4L* (mut) were re-overexpressed (Fig. 4*D*). *ORP4L* knockdown completely prevented OCRL and PLCβ3 translocation, therefore we found an intense PI(4,5)P_2_ accumulation at the PM in the *ORP4L* knockdown cells with or without anti-CD3 stimulation. Re-overexpression of the wildtype *ORP4L* abolished the PI(4,5)P_2_ accumulation, while the *ORP4L* (mut) only partly rescued this phenotype (Figs. 4, *E* and *F* and S3*B*). These results suggested that PI(4,5)P_2_ at the PM was consumed by OCRL and PLCβ3, both of them dependent of ORP4L.

### OCRL is essential for cytosol and mitochondria Ca^2+^ homeostasis of Jurkat T-cells

**Figure 5:**
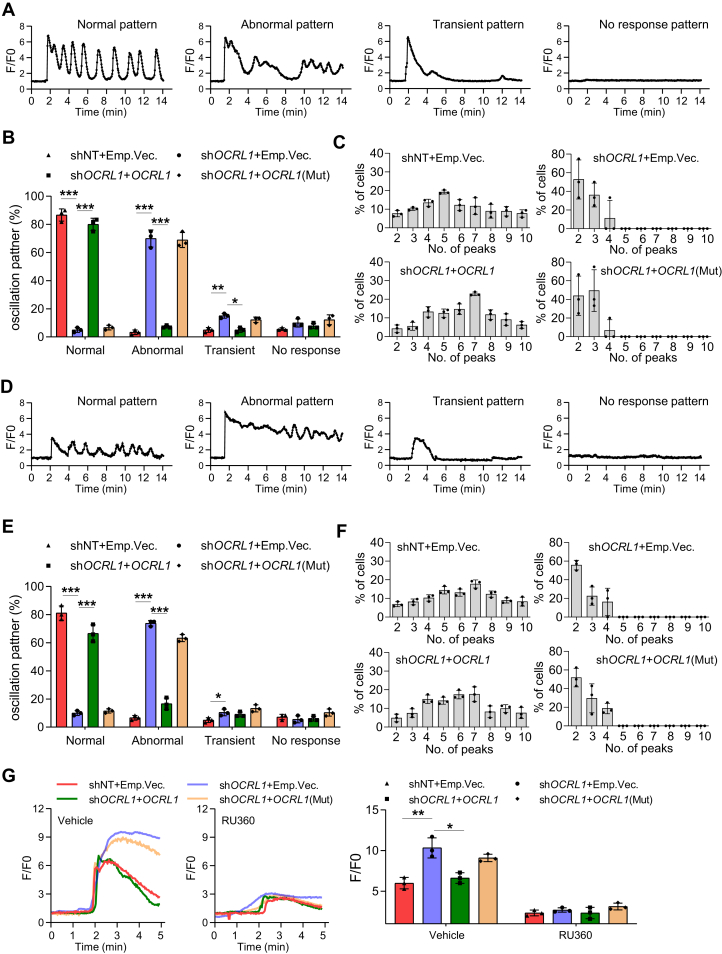
**OCRL modulates cytosolic and mitochondrial Ca**^**2+**^**homeostasis in Jurkat T-cells.***A*, the four types of cytosolic calcium oscillation patterns in Jurkat T-cells upon low concentration of anti-CD3 (1 μg ml^−1^) stimulation. *B*, the percentage of cells represent cytosolic calcium oscillation patterns from three independent experiments. Changes in [Ca^2+^] were recorded as F/F0 ratio using Fluo-4-AM in confocal Ca^2+^ imaging. For each experiment, >30 cells of each group were quantified. *C*, the percentage of cells represent different number of calcium peak in cells with normal oscillation pattern. *D*, the four types of mitochondrial calcium oscillation patterns in Jurkat T-cells upon low concentration of anti-CD3 (1 μg ml^−1^) stimulation. Changes in [Ca^2+^] were recorded as F/F0 ratio using Rhod-2 in confocal Ca^2+^ imaging. *E*, the percentage of cells represent mitochondrial calcium oscillation patterns from three independent experiments. For each experiment, >30 cells of each group were quantified. *F*, the percentage of cells represent different number of calcium peak in cells with normal oscillation pattern. *G*, high concentration of anti-CD3 (10 μg ml^−1^)-induced [Ca^2+^] peaks in mitochondrial of Jurkat T-cells with or without MCU inhibitor RU360 pretreatment (5 μM, 30 min). Changes in [Ca^2+^] were recorded as the F:F0 ratio using Rhod-2 in confocal Ca^2+^ imaging. Average [Ca^2+^] peak amplitudes were shown from three independent experiments. For each experiment, >30 cells of each group were quantified. The data represent mean ± SD (n = 3). ∗*p* < 0.05, ∗∗*p* < 0.01, ∗∗∗*p* < 0.001; Student's *t* test. CD3, cluster of differentiation 3; MCU, mitochondrial Ca2+ uniporter; OCRL, oculocerebrorenal syndrome of Lowe 1.

Ca^2+^ signaling has been closely associated with PLC-mediated PI(4,5)P_2_ breakdown, namely through IP_3_-induced Ca^2+^ release from the ER (15). Therefore, we measured the cytosol Ca^2+^ oscillation in *OCRL1* knockdown Jurkat T-cells after low concentration of anti-CD3 stimulation. To analyze results quantitatively, we classified intracellular Ca^2+^ signal patterns into four groups (Fig. 5*A*): normal oscillation pattern consists of repetitive spike-shape Ca^2+^ rises which interval are about 2 min; abnormal oscillation consists of a combination of continuous Ca^2+^ rise and oscillation; transient pattern consists of one transient Ca^2+^ rises; no-response pattern consists of no Ca^2+^ rises during observation periods. We found most of the control cells displayed normal oscillation pattern upon anti-CD3 stimulation, and *OCRL1* knockdown decreased the normal pattern responding cells, but increased the abnormal and transient pattern responding cells (Fig. 5*B*). In rescue experiments, *OCRL1* but not *OCRL1*(mut) re-overexpression abolished the reduced normal pattern resulting from *OCRL1* knockdown (Fig. 5*B*). We quantified frequency of normal pattern oscillation. The number of peaks seen during stimulation was 2 to 10 peaks in control cells, but there were only 2 to 4 peaks in *OCRL1* knockdown cells, the reduction can be rescued by *OCRL1*, but not the *OCRL1*(mut) (Fig. 5*C*).

Constitutive Ca^2+^ oscillations released periodically from the ER are taken up by mitochondria *via* the MCU (17), and T-ALL cells had mitochondrial Ca^2+^ oscillation follow the same patterner as cytosol Ca^2+^ oscillation (32). We next analyzed the mitochondrial Ca^2+^ oscillation patterns. *OCRL1* knockdown also decreased the mitochondrial normal pattern and the number of peaks, which can be rescued by *OCRL1*, but not the *OCRL1*(mut) (Fig. 5, *D*–*F*). To study whether the amount of PI(4,5)P_2_ affects the Ca^2+^ signal patterns, we pretreated Jurkat T-cells with exogenous PI(4,5)P_2_, the moderate addition of PI(4,5)P_2_ increased the normal oscillation pattern and the number of peaks. Whereas, the excess PI(4,5)P_2_ produces opposite effect and results in decreased normal pattern and number of peaks but increased abnormal pattern (Fig. S4, *A* and *B*).

Excessive Ca^2+^ taken up by MCU into mitochondria results in mitochondrial Ca^2+^ overload. To strengthen the link between OCRL function and mitochondrial, we next assayed Ca^2+^ uptake by mitochondrial by MCU. *OCRL1* knockdown markedly increased the Ca^2+^ peak amplitude in mitochondria (Fig. 5*G*) in high concentration of anti-CD3–stimulated cells, whereas *OCRL1* but not *OCRL1* (mut) re-overexpression reduced this amplitude (Fig. 5*G*). To test whether *OCRL1* knockdown induces mitochondrial Ca^2+^ overloading *via* MCU complex, we blocked the pore with the MCU inhibitor Ruthenium 360 (Ru360). The highly peak amplitude of *OCRL1* knockdown cells was significantly reduced in Ru360 pretreated cells (Fig. 5*G*). Altogether, these results suggest that *OCRL1* knockdown leads to excess PI(4,5)P_2_ accumulation in PM and cytosol and mitochondrial Ca^2+^ dyshomeostasis in T-ALL cells.

### OCRL sustains mitochondrial function and T-ALL cell survival *in vivo*

**Figure 6:**
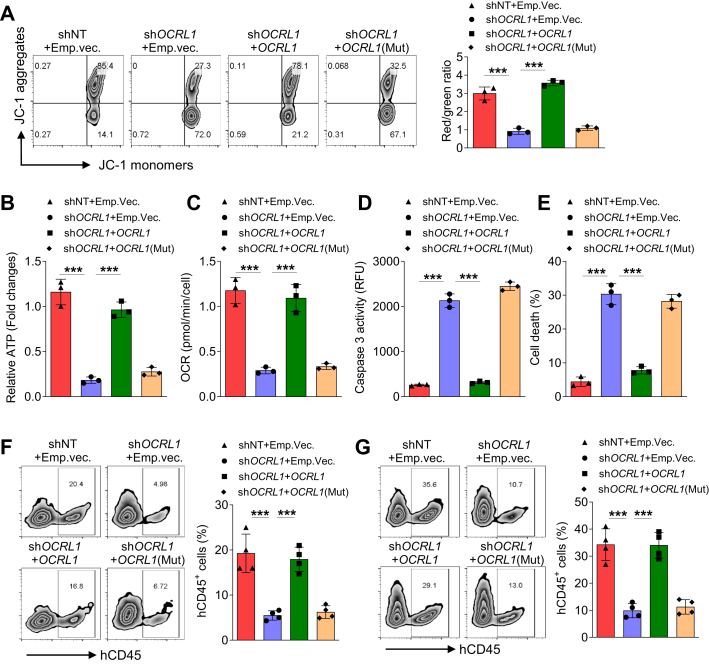
**OCRL regulates mitochondrial function and cell survival *in vivo*.***A*, flow cytometry analysis of mitochondrial membrane potential by JC-1 staining in Jurkat T-cells transduced with the indicated lentiviruses. The histograms expressed as ratio of JC-1 aggregates (*red*) and monomer (*green*) (*Red/Green*). *B*–*E*, the ATP levels (*B*), OCR (*C*), caspase-3 activity (*D*) and cell death rate (*E*) of Jurkat-T cells transduced with the indicated lentiviruses. *F* and *G*, flow cytometric analysis of engrafted Jurkat T-cells in the bone marrow (*F*) and spleen (*G*) of NSG mice. Jurkat T-cells transduced with the indicated lentiviruses were injected *via* the tail vein, and after 6 weeks, the mice were sacrificed for analysis. The data represent mean ± SD (n = 3 for *A*–*E*, n = 4 for *F* and *G*). ∗∗∗*p* < 0.001; Student's *t* test. OCR, oxygen consumption rate; OCRL, oculocerebrorenal syndrome of Lowe 1.

Mitochondrial Ca^2+^ overload results in mitochondrial permeability transition pore opening, release of cytochrome c, activation of caspase3/7, and ATP synthesis inhibition (19). We next asked whether the Ca^2+^ overloading in mitochondrial have a detrimental effect and lead to cell death. *OCRL1* knockdown decreased the mitochondrial membrane potential as measured by JC-1 (Fig. 6*A*), decreased ATP production and oxygen consumption rate (Fig. 6, *B* and *C*), and also increased the Caspase 3 activity and accelerated cell death (Fig. 6, *D* and *E*). These phenotypes were all rescued by wildtype *OCRL1* but not *OCRL1* (mut) re-overexpression (Fig. 6, *A*–*E*).

A xenograft model was established in NSG mice to determine the contribution of OCRL to T-ALL cell survival *in vivo*. Mice were injected with Jurkat T-cells with OCRL1 gene modifications. At 6 weeks postinjection, the mice were scarified, and the engraftment of T-ALL cells were detected. We found the percentage of engrafted cells in bone marrow and spleen was significantly lower in mice injected with *OCRL1* knockdown cells. Whereas, this reduction was abolished only in mice injected with *OCRL1* re-overexpression cells, but not the *OCRL1*(mut) re-overexpression cells (Fig. 6, *F* and *G*).

## Discussion

Previous studies have shown that OCRL in Lowe syndrome patient cells translocates from the Golgi to the PM after Rac activation, and its deficiency causes PI(4,5)P_2_ to accumulate at the PM (6). This is a new perspective on Lowe syndrome pathophysiology. It demonstrates that the Golgi/trans-Golgi network is not the only organelle affected by loss of OCRL function and raises the question of the role of OCRL-induced PM perturbation in the pathophysiology of Lowe syndrome. According to this finding, an anomalous mode of OCRL action may exist in some diseases in which *OCRL1* expression is misregulated. In this study, we found that OCRL interacts specifically with ORP4L, and anti-CD3 stimulation enhances the colocalization between OCRL and ORP4L, facilitating more OCRL to be translocated from the Golgi to the PM in T-ALL cells. Confocal imaging showed the redistribution of OCRL and colocalization with ORP4L at the PM. This phenomenon occurs only when the interaction sites of OCRL and ORP4L remain intact. These findings suggest that OCRL anchors to ORP4L upon translocation from the Golgi to the PM.

As an inositol polyphosphate 5-phosphatase that dephosphorylates PI(4,5)P_2_, OCRL plays an important role in controlling the PI(4,5)P_2_ content of membranes. Translocation of OCRL increases hydrolysis of PI(4,5)P_2_ at the membrane, converting it to PI(4)P, thereby maintaining the balance between PI(4,5)P_2_ and PI(4)P (33). When *OCRL1* was knocked down, the balance was broken and PI(4,5)P_2_ abundantly accumulated at the PM. In addition, PI(4,5)P_2_ is a precursor of IP_3_ and diacylglycerol after PLC activation, which is essential for early signaling of cell surface receptors. ORP4L plays an important role in this process (24). PLC is activated upon anti-CD3 stimulation (23), and the accumulation of PI(4,5)P_2_ results in a corresponding increase in the second messenger IP_3_. Similar to our observation in *OCRL1* knockdown cells, glutamate regulates Ca^2+^ oscillations in a concentration-dependent manner, low concentration increases oscillation frequency, but high concentration disrupts oscillation and results in single high amplitude (34). Mitochondrial Ca^2+^ is a double-edged sword. The low levels of Ca^2+^ is required for ATP production, but extreme level Ca^2+^ leads to loss of mitochondrial. Thus, the moderate amount of PI(4,5)P_2_ in PM used for hydrolysis is important for IP_3_/Ca^2+^ signaling and cell survival. *OCRL1* knockdown resulting in excessive PI(4,5)P_2_ hydrolysis by PLCβ3 leads to excessive ER Ca^2+^ release and disruption of constitutive Ca^2+^ oscillation pattern and cell death.

In recent years, great progress has been made in understanding the role played by OCRL in cellular metabolism. OCRL appears to regulate many processes within the cell, most of which depend on the coordination of membrane dynamics with actin cytoskeleton remodeling (35). Here, we identify a previously unknown translocation mechanism of OCRL in T-ALL cells that may influence T-ALL progression. This finding is novel, but further studies are needed to determine the importance of OCRL in T-ALL cells and the uniqueness of this translocation mechanism of OCRL in T-ALL cells. Because of the difference in expression between normal T-cells and T-ALL cells, we connected OCRL to another protein, ORP4L, which plays an important role in T-ALL and confirmed their interaction. Another question is whether there is an alternative enzyme to OCRL in T-ALL cells. The inositol polyphosphate 5-phosphatase INPP5B is closely related to OCRL, sharing a similar substrate specificity, domain organization, overlapping subcellular distribution and similar interaction partners (36). In particular, OCRL and INPP5B are the only human 5-phosphatases with a RhoGAP-like domain, both widespread in vertebrates (37). Determining whether INPP5B affects the physiologic effects of OCRL depletion is one of the necessary topics of follow-up research. Finally, as OCRL appears as a new factor regulating energy metabolism in T-ALL, its possible use as a target for T-ALL treatment needs further verification by *in vivo* experiments.

In conclusion, in this study, we revealed that OCRL controls the activity of ORP4L upon translocation from the Golgi to the PM, by consuming PI(4,5)P_2_ together with PLCβ3 to maintain Ca^2+^ homeostasis. The role of OCRL in T-ALL cells survival suggests that it may be a viable therapeutic target for T-ALL, providing a new idea to the treatment of this deadly malignancy.

## Experimental procedures

### Human leukocyte specimens and cell lines

The human leukocytes and cell lines used were cultured as described in our previous study (23). Briefly, peripheral blood mononuclear cells from healthy human donors and T-ALL patients were isolated by Ficoll-Hypaque gradient centrifugation, followed by purified using an Enhanced Human T Cell Recovery Column Kit (Cedarlane). The Jurkat and Molt-4 cells were maintained in RPMI1640 containing 10% FBS, 100 U/ml penicillin, and 100 mg/ml streptomycin at 37 °C in a humidified incubator with 5% CO_2_.

### Cell transfection

High-titer lentivirus (>10^9^ transduction units per mL) expressing nontargeting shRNA (shNT), *OCRL1*-specific shRNA (sh*OCRL1*, sh*OCRL1*#2), *ORP4L* shRNA (sh*ORP4L*), or the *OCRL1* cDNA, *OCRL1*(mut) cDNA, *ORP4L* cDNA, and the *GFP-PH*_*PLCδ1*_ domain cDNA were packaged by Shanghai GenePharma (Shanghai,China). The shRNA targeting sequences used were: *ORP4L*: 5′-TCAGAGTCAAGCTCAGGTGTA-3′; *OCRL1*: 5′-GGTTCCCTGCCATTTTTCA-3′; *OCRL1*#2: 5′-TGAGAGAGCGCCGCTTTGA-3′. For single lentivirus transduction, cells were transduced and culture for 72 h before used. For two separate lentivirus transductions, cells were transduced with the shRNA lentivirus and cultured for 48 h, followed by the cDNA lentivirus transduction and culturing for additional 48 h. For lentivirus infection, 1 × 10^5^ cells were resuspended in 100 μl of medium supplemented with polybrene (5 μg/ml) and containing lentivirus [multiplicity of infection = 100] in 24-well culture plates. Spinfection was conducted by spinning the plates at 250 g for 1 h at 25 °C. Then 400 μl of medium was added dropwise on top of each well and cultured at 37 °C.

### Quantitative RT-PCR

Total RNA was isolated with TRIzol Reagent (Invitrogen) according to the manufacturer’s instructions. RNA samples were reverse transcribed using random hexamer primers in the presence of RNase Inhibitor (Takara Bio). Quantitative RT-PCR was performed with SYBR Premix EX Taq (Takara Bio) using the CFX96 Sequence Detection System (Bio-Rad Laboratories). A relative quantification analysis was performed using the ΔΔ cycle threshold method, and the expression levels were normalized using actin as endogenous control. Relative gene expression is presented as log (2^−ΔΔ^ cycle threshold). The primer sequences are as following: *ORP4L* (sense 5′-CCCTTCACTAAGGCCGCATC-3′, anti-sense 5′-GAACCCCAAGAGGAGTCTTCG-3′); *OCRL1* (sense 5′-CACTGACCTGGGATCTTTG-3′, anti-sense 5′-CCAGCTGAATCCGAAATCC-3′); *Actin* (sense 5′-GGCATCCTCACCCTGAAGTA-3′, anti-sense 5′-AGGTGTGGTGCCAGATTTTC-3′).

### Cell proliferation and cell death assay

Cell proliferation was analyzed by using the Click-iT Plus EdU Alexa Fluor 488 Flow Cytometry Assay Kit. Cells were treated with 10 μM EdU for 1 h and stained with Invitrogen Alexa Fluor 488 picolyl azide, according to the protocol for the Invitrogen Click-iT Plus EdU Alexa Fluor 488 Flow Cytometry Assay Kit. Cells were then analyzed by flow cytometry using 488 nm excitation (for Click-iT EdU Alexa Fluor 488 dye).

Cell death was analyzed by LIVE/DEAD Fixable Dead Cell Stain Kits (Life Technologies) according to the manufacturer's instructions. Briefly, cells were washed once with PBS and then incubated with LIVE/DEAD Fixable Dead Cell Stain in PBS for 30 min at room temperature in the dark. After washing with PBS with 1% FBS, cells were resuspended in PBS with 1% FBS and analyzed using flow cytometer (FACSAriaTM, BD), and the data were analyzed by FlowJo_V10 software.

### Coimmunoprecipitation

Cells were washed twice with ice-cold PBS and incubated on ice for 30 min with 1 ml of lysis buffer (50 mM Tris-Cl, 150 mM NaCl, 0.5 mM MgCl_2_, 10% glycerol, and 0.5% Triton X-100, pH 8.0) supplemented with protease inhibitor mixture (Roche Applied Science). Cell lysates were centrifuged for 15 min at 15,000*g*. The supernatant was preabsorbed with 50 μl of Protein G-agarose (Invitrogen) for 1 h at 4 °C. The recovered supernatant was incubated overnight with OCRL1 or control antibody at 4 °C. 50 μl of Protein G-agarose was added to the lysate–antibody mixture and incubated for 2 h at 4 °C on a roller. The beads were washed four times with lysis buffer and boiled in SDS-PAGE loading buffer. Samples were resolved on 10% SDS-polyacrylamide gels and subjected to Western blot analysis with antibodies.

### GST pulldown assay

The wildtype *ORP4L*, *OCRL1*, *OCRL1*(mut), or *ORP4L*(mut) cDNA were cloned into pGEX-4T-1 vector. These constructs were transformed into *E. coli* RosettaTM (DE3) (Novagen) and cultured at 37 °C to A600 = 0.5 to 1.0, followed by induction with 0.1 mm isopropyl 1-thio-β-d-galactopyranoside to induced protein expression for 16 to 18 h at 18 °C. The cells were collected, and crude bacterial lysates were prepared by sonication in lysis buffer 1 (50 mM Tris-Cl, 150 mM NaCl, 1% Triton X-100, and 1 mM phenylmethylsulfonyl fluoride, pH 8.0) in the presence of the protease inhibitor mixture (Roche Applied Science). Bacterial lysates were centrifuged for 20 min at 12,000*g*, the soluble protein supernatants were collected, and the protein concentration detected by using BCA Protein Assay Kit (Beyotime). Approximately equal amounts of different GST fusions were incubated with glutathione-Sepharose 4B beads (GE Healthcare) for 1 h at 4 °C, then the samples were centrifuged at 1300 g for 1 min to collect GST protein-beads complexes. For pulldown, Jurkat T-cells were washed twice with cold PBS, lysed in lysis buffer, and shaken for 30 min on ice, and the lysate was cleared by a 10-min centrifugation at 12,000 rpm in a microcentrifuge. Jurkat T-cells lysates were then added and incubated with GST protein–beads complexes at 4 °C overnight. Then the beads were washed three times with lysis buffer, resuspended into 2 × SDS-PAGE loading buffer at 98 °C for 5 min, and resolved on 10% SDS-polyacrylamide gels for Western blotting. Approximately equal amounts of different GST fusions used were verified by immunoblotting with anti-GST antibody.

### Immunofluorescence microscopy

For protein staining, cells were seeded onto coverslips, stimulated with anti-CD3 for the indicated times, and then fixed with 4% paraformaldehyde for 30 min at room temperature followed by permeabilization with 0.1% Triton X-100 for 5 min and blocking with 10% FBS for 30 min at room temperature. Cells were then incubated with primary antibodies in 5% FBS at 4 °C overnight. After washing three times (10 min each) with PBS, cells were incubated with fluorescent secondary antibody conjugates at 37 °C for 30 min followed by staining with Hoechst 33342 at room temperature for 10 min. Antibodies are used as following: Rabbit anti-ORP4L (Sigma-Aldrich, 1:200), mouse anti-OCRL (Santa Cruz, 1:50), Alexa Fluor 488-Goat Anti-Mouse IgG (H + L) secondary antibody (Thermo Fisher Scientific, 1:200), and Alexa Fluor 546 Goat Anti-Rabbit IgG (H + L) secondary antibody (Thermo Fisher Scientific, 1:200). The specimens were analyzed using Olympus FV3000 laser-scanning confocal microscope system.

### Yeast 2-hybrid analysis

To identify the interaction regions of OCRL and ORP4L, full-length *OCRL1* or *ORP4L* in bait vector pGBKT7 and the truncated fragments of ORP4L or OCRL1 in prey vector pGADT7 (Takara Bio) were cotransformed into the yeast stain AH109 before plating on synthetic defined medium without Leu, Trp (SD/2-) and without Ade, His, Leu, and Trp (SD/4-) plates and culturing at 30 °C. The colonies that appeared were then transferred onto membrane for X-gal assay. The interaction was monitored as growth on SD/4- and by X-gal cleavage assay. The vectors pGBKT7-53 and pGADT7-T were used as positive controls because the murine p53 protein (53) can interact with SV40 large T-antigen (T) in yeast, while pGBKT7-Lam and pGADT7-T were used as negative controls because human lamin C protein (Lam) cannot interact with T in yeast. The empty plasmid pGBKT7 was used to exclude self-activating activity.

### Homology modeling and docking simulation

The structure prediction of partial ORP4L was generated using Rosetta (38, 39). Hydrogen atoms were added to the protein using the MOE (MOE 2015.10. Chemical Computing Group, Inc.) before carrying out the docking studies. The structures of ORP4L and OCRL (PDB: 2QV2) were subjected to an energy minimization protocol. The atomic partial charges were calculated with the Amber12:EHT force field, and all possible ionization states were generated at pH 7.0 using the MOE suite. ORP4L was docked into OCRL using MOE software. The top scoring conformation was used for the binding model analysis.

### *In vitro* phosphatase activity assays

Cells were incubated with 1 μM soluble short-chain diC8-PI(4,5)P_2_ (Echelon Biosciences) for 10 min at room temperature in culture medium, then the PM was prepared by using Plasma Membrane Protein Isolation and Cell Fractionation Kit (Invent Biotechnologies, Inc.) according to the manufacturer’s instructions. The assay mixture consisted of 50 μl of assay buffer (50 mM Hepes, pH 7.0, 100 mM KCl, 6 mM MgCl_2_, 0.6 mM CaCl_2_, 2 mM EGTA), and 10 μg of PM was incubated on ice for 10 min. The reaction was started by the addition of 5 μg of OCRL or OCRL(mut) recombinant proteins and incubation at 37 °C for 10 min. After the reaction, The PM pellet was centrifuged and collected for analysis of PI(4)P and PI(4,5)P_2_ levels by dot blot.

### BiFC assay

BiFC constructs using fragments derived from Venus were generated, and wildtype or mutated *ORP4L* and *OCRL1* cDNAs were inserted into pVn-N1, pVc-N1, pVn-C1 and pVc-C1 vectors (40). The *OCRL1*/pVn-N1 and *ORP4L*/pVc-N1, *OCRL1*/pVn-C1 and *ORP4L*/pVc-C1, *OCRL1*/pVn-N1 and *ORP4L*/pVc-C1, *OCRL1*/pVc-N1 and *ORP4L*/pVn-C1, or *OCRL1*(Mut)/pVn-N1 and *ORP4L*/pVc-C1 vectors were cotransfected into Jurkat T-cells by using an 4D-Nucleofector System (Lonza) according to the manufacturer’s instructions. After cultured for 24 h, cells were stimulated with or without 10 ug/ml anti-CD3 for 5 min and then fixed with 4% paraformaldehyde for 15 min at 4 °C. The BiFC fluorescence was detected by using Olympus FV3000 confocal Microscope.

### Isolation of Golgi and PM fractions

PM and Golgi fractions were prepared by using Plasma Membrane Protein Isolation and Cell FractionationKit (Invent Biotechnologies, Inc) and Golgi Apparatus Enrichment Kit (Invent Biotechnologies, Inc) according to the manufacturer’s instructions.

### Dot blots for PI(4,5)P_2_ and PI(4)P levels

PI(4,5)P_2_ and PI(4)P of PMs were released by incubation with lysis buffer (50 mM Tris, pH 7.5, 300 mM NaCl, 5 mM EGTA, 20 mM DTT, 2% Triton X-100 and 50 mM NaF) at room temperature for 30 min. Dot blots were conducted as described (41). Briefly, after centrifugation, 1 μl of suspension was spotted onto nitrocellulose membrane (Bio-Rad Laboratories), probed with PI(4,5)P_2_ antibody (1:500, Echelon Biosciences), PI(4)P antibody (1:500, Echelon Biosciences), or PM internal loading control pan-cadherin antibody (Santa Cruz), and detected using an HRP-conjugated secondary antibody and enhanced chemiluminescence. Experiments were repeated at least three times.

### Ca^2+^ oscillation and amplitude measurement

Cells (1 × 10^5^) were incubated with 1 μM Fluo-4 AM (for cytosolic Ca^2+^ measurement) or 2 μM Rhod-2 AM (for mitochondrial Ca^2+^ measurement) for 15 min at 37 °C in extracellular calcium buffer (130 mM NaCl, 5 mM KCl, 1.5 mM CaCl_2_, 1 mM MgCl_2_, 25 mM Hepes, pH 7.5, 1 mg/ml BSA, and 5 mM glucose) in dark, after which they were collected and resuspended in extracellular calcium buffer for an additional incubation at 25 °C for 30 min to permit dye de-esterification. Then, cells were plated onto a glass-bottomed dish and excited with low-intensity 488-nm laser excitation (for Fluo-4 AM) or 516-nm laser excitation (for Rhod-2 AM). Images were acquired at 3-s intervals under time-lapse mode by Olympus FV3000 confocal Microscope. For oscillation measurement, fluorescence was collected for 1 min before the low concentration of anti-CD3 (1 ug/ml) was added into suspension. For amplitude measurement, fluorescence was collected for 1 min before the high concentration of anti-CD3 (10 ug/ml) was added into suspension. Image data were subsequently analyzed using ImageJ (National Institutes of Health) and are presented as a ratio of F/F0 in final results, where F0 represents baseline fluorescence intensity in each cell.

### Mitochondrial membrane potential measurement

The mitochondrial membrane potential was detected by using JC-1 dye (Beyotime). 5 × 10^5^ cells were suspended in 0.5 ml PBS containing 1 μM JC-1 and incubated for 15 min at 37 °C in dark. After washed once with PBS, the green and red fluorescence were detected by flow cytometer (FACSAriaTM, BD). The green fluorescence represents JC-1 monomers, whereas red fluorescence represents JC-1 aggregates.

### Oxygen consumption assay

Oxygen consumption was analyzed by using MitXpress-Xtra-HS (Cayman Chemical). 2 × 10^5^ cells were transferred into fresh culture medium containing 1% FBS, then 10 μl of porphyrin-based phosphorescent oxygen-sensitive probe was added, and the cells were equilibrated at 37 °C. The assay was read by a microplate reader. The maximal rate of oxygen consumption is proportional to the change in probe fluorescence during the linear phase of the assay.

### ATP measurement

ATP levels were measured using ATP Assay Kit (Abcam). All the reagents such as ATP standard dilution, ATP reaction mix, and Background reaction mix were prepared according to manufacturer’s protocol. ATP standards were prepared to obtain standard curve. 1 × 10^6^ cells were seeded into 6-well plates and lysed with lyolysis on ice. The samples were centrifuged for 5 min at 13,000*g* to remove insoluble material. The supernatants were collected and transferred to new tubes. Before loading on 96-well plate, volume of all samples was adjusted to 50 μl with ATP Assay Buffer. Reaction mix (50 μl) was added into each standard and sample well, and 50 μl of background reaction mix was added into the background control sample wells. The plate was incubated at room temperature for 30 min protected from light. Absorbance was detected at 570 nm using a microplate reader. The results are presented as the ratio between the test and control values.

### Fluorogenic caspase 3 activity assay

1 × 10^6^ cells were washed with PBS and lysis by cell lysis buffer (Cell Signaling Technology). After centrifuged at 4 °C for 10 min at 12,000*g*, cell lysates were diluted to a concentration of approximately 5 μg/μl. Caspase activity assay (Cell Signaling Technology) was performed according to the manufacturer’s instructions. Fluorescence was detected by excitation of 380 nM and emission of 460 nm and expressed in relative fluorescence units.

### Human T-ALL cell xenograft *in vivo*

The 4- to 6-weeks-old female NSG mice (Beijing Biocytogen) were kept under pathogen-free conditions in the Laboratory Animal Center, Jinan University. All animal procedures were conducted in accordance with the Guidelines for the Care and Use of Laboratory Animals and were approved by the Laboratory Animal Ethics Committee at Jinan University. To establish an engraftment model, Jurkat T-cells were transfected with lentivirus and cultured for 48 h, then injected (2 × 10^7^ cells/0.2 ml of PBS) *via* the tail vein. Weekly monitoring of mice for circulating leukemia cells in peripheral blood was performed by analysis of human CD45 expression with flow cytometry. At 6 weeks postengraftment, mice were killed. Cells were then isolated from spleens by mechanical disaggregation and red blood cell lysis and collected from bone marrow by flushing of femurs with PBS. The leukemia cells burden in spleens and bone marrow were staining with CD45 antibody and assessed by flow cytometer (FACSAriaTM, BD).

### Western blot analysis

Cells were suspended in lysis buffer (50 mM Tris-Cl, pH8.0, 150 mM NaCl, 0.5 mM MgCl_2_, 10% glycerol, 1% Triton X-100, 0.1% SDS) with protease inhibitor cocktail (Roche Diagnostics) on ice for 30 min. Samples were centrifuged at 4 °C for 10 min at 12,000*g*. The supernatants were collected, and protein concentrations were measured using the BCA Protein Assay Kit (Beyotime) according to the manufacturer’s instructions. Protein extracts were run on a 10 or 12% SDS polyacrylamide gel before they were transferred to a polyvinylidene difluoride membrane. Membranes were blocked with 5% milk for 1 h and incubated with primary antibodies overnight, followed by incubation with the secondary antibodies for 1 h at room temperature. Antibodies are used as following: anti-ORP4L (Sigma-Aldrich, 1:1000), anti-OCRL (Santa Cruz, 1:200), anti-pan-cadherin (Santa Cruz, 1:200), anti-GM130 (Thermo Fisher Scientific, 1:3000), anti-Canexin (Cell signaling, 1:1000), anti-Cytochrome c (Santa Cruz, 1:200), anti-p-PDH (Novus Biologicals, 1:100), anti-PDH (Cell signaling, 1:1000), anti-actin (Proteintech Group, 1:3000), Goat anti-rabbit IgG (H + L), HRP conjugate antibody (Proteintech Group, 1:3000), Goat anti-mouse IgG (H + L), and HRP conjugate antibody (Proteintech Group, 1:3000).

### Statistical analyses

The data were expressed as mean ± SD from at least three independent experiments. All comparisons between groups were made by unpaired two-tailed Student's *t* test. *p* values of <0.05 were considered statistically significant.

## Data availability

All data generated for this study are included within this article.

## Supporting information

This article contains supporting information.

## Conflict of interest

The authors declare that they have no conflicts of interest with the contents of this article.
