# Supplementary material for: Oculocerebrorenal syndrome of Lowe (OCRL) controls leukemic T-cell survival by preventing excessive PI(4,5)P2 hydrolysis in the plasma membrane
Source: J Biol Chem. 2023 May 11;299(6):104812. doi: 10.1016/j.jbc.2023.104812 (PMC10279916; doi:10.1016/j.jbc.2023.104812)
Supplement: Supporting information [file mmc1.pdf]

Supporting information

Oculocerebrorenal syndrome of Lowe (OCRL) controls leukemic T-cell survival by preventing excessive $PI(4,5)P_2$ hydrolysis in the plasma membrane

Huanzhao Chen[1], Chen Lu[1], Yuhui Tan[1], Marion Weber-Boyvat[2,3], Jie Zheng[1], Mengyang Xu[1], Jie Xiao[1], Shuang Liu[1], Zhiquan Tang[1], Chaofeng Lai[1], Mingchuan Li[1,4], Vesa M. Olkkonen[2], Daoguang Yan[1,4*], Wenbin Zhong[1,4*]

Contents

Supporting Figures S1-S4

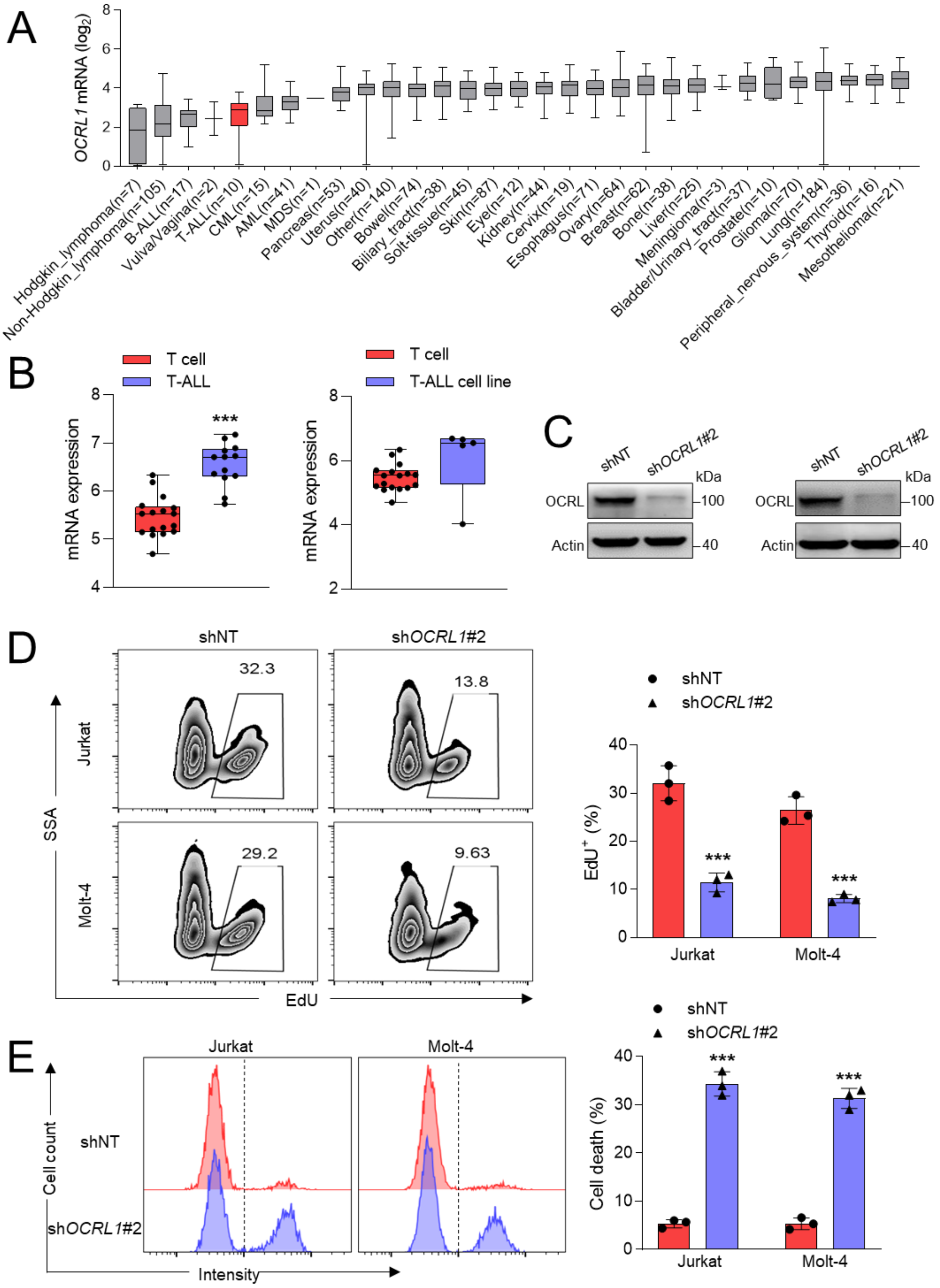

Figure S1. ***OCRL1*** **is highly expressed in T-ALL cells and its depletion impairs cell survival.** *A, OCRL1* expression was analyzed among 1036 human cancer cell lines in CCLE database (https://portals.broadinstitute.org/ccle). *B, OCRL1* expression levels in normal T cells (n=17) and primary T-ALL patients cells (n=13) and T-ALL cell lines (n=5) from GEO database, GSE48558. *C*, western blot showing OCRL expression in Jurkat T-cells (left) and Molt-4 cells (right) subjected to control (shNT) and *OCRL1* knockdown (sh*OCRL1*#2). Cells were transduced with lentivirus expressing control

non-targeting shRNA or *OCRL1* specific shRNA and culture for 96h. *D*, cell proliferation analysis of Jurkat T-cells and Molt-4 cells subjected as in panel *C*. Cells were incubated with EdU for 1 h and analyzed by flow cytometer. *E*, cell death analysis of Jurkat T-cells and Molt-4 cells subjected as in panel *C*. Cells were stained by LIVE/DEAD Fixable Dead Cell Stain Kits and analyzed by flow cytometer. The data represent mean ± SD (n=3). ***P < 0.001; Student's *t* test.

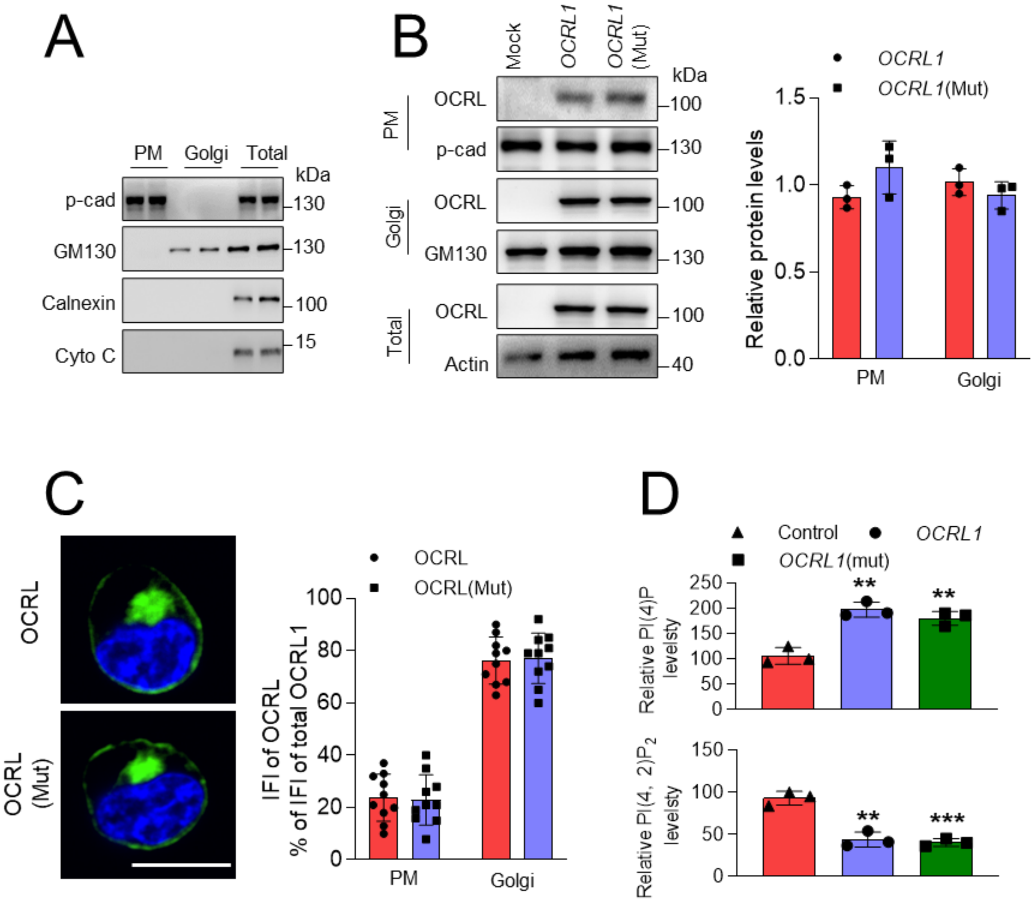

Figure S2. The OCRL (mut) protein localization and phosphatase activity assay. *A*, western blot showing the distribution of p-cad, GM130, Calnexin and Cyto C in PM, Golgi and total lysate of Jurkat T-cells. *B*, western blot showing the distribution of OCRL, OCRL (mut) in PM, Golgi and total lysate of Jurkat T-cells. OCRL were detected by Xpress antibody. The histograms showed the relative OCRL protein levels in PM and Golgi from 3 independent experiments. *C,* immunofluorescence staining showing the OCRL and OCRL (mut) intracellular localization in Jurkat T-cells. OCRL were detected by Xpress antibody. The histograms showed the immunofluorescence intensity (IFI) in PM and Golgi of total IFI from n=10 cells for each group. Scale bar, 10 μm. *D,* the PI(4)P production and PI(4,5)P_2 reduction in PM of *in vitro* phosphatase assay. Recombinant OCRL and OCRL (mut) protein were incubated with PM, phosphatase activity was measured as described in the Experimental procedures. The data represent mean ± SD (n=3). **P < 0.01; ***P < 0.001; Student's t test.

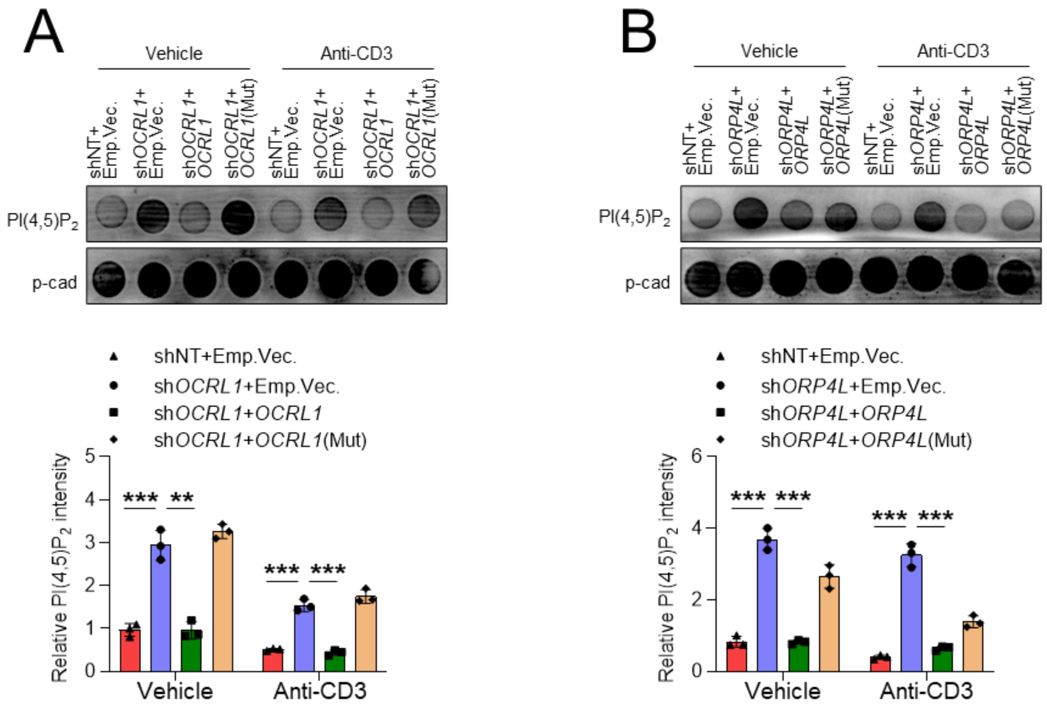

Figure S3. Dot blot detected PI(4,5)P2 levels at the PM. *A,* The cells were subjected to control (shNT), *OCRL1* knockdown (sh*OCRL1*) alone, or combined with *OCRL1* rescued expression (sh*OCRL1*+*OCRL1*), or combined with *OCRL1*(Mut) rescued expression (sh*OCRL1*+*OCRL1*(Mut)). Cells were transduced with lentivirus expressing shNT or sh*OCRL1* for 48h, before transduction with lentivirus expressing *OCRL1* or *OCRL1*(Mut). After additional 48h of culture, the cells were stimulated with 10 μg ml^{-1} anti-CD3 for 5 min before PM preparation. *B,* The cells were subjected to control (shNT), *ORP4L* knockdown (sh*ORP4L*) alone, or combined with *ORP4L* rescued expression (sh*ORP4L*+*ORP4L*), or combined with *ORP4L* (Mut) rescued expression (sh*ORP4L* + *ORP4L* (Mut)). Cells were transduced with lentivirus expressing shNT or sh*ORP4L* for 48h, before transduction with lentivirus expressing *ORP4L* or *ORP4L* (Mut). After additional 48h of culture, the cells were stimulated with 10 μg ml−1 anti-CD3 for 5 min before PM preparation. PI(4,5)P2 levels expressed as fold change are shown. The data represent mean ± SD (n=3). $^{**}P < 0.01$, $^{***}P < 0.001$; Student's t test.

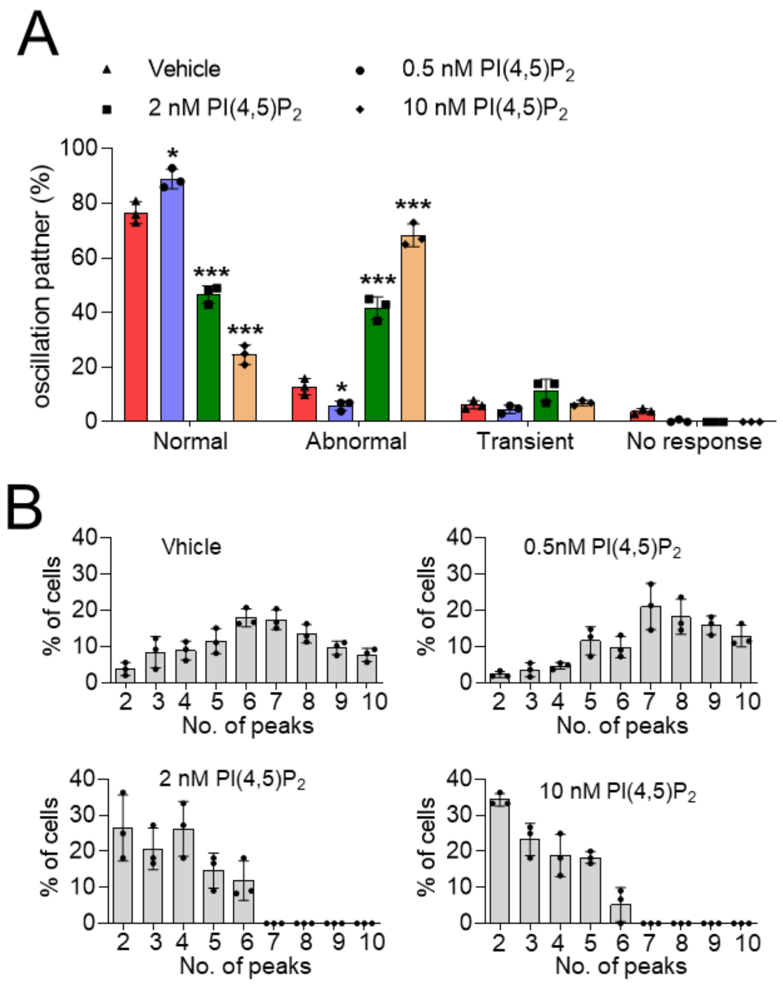

Figure S4. The role of exogenous PI(4,5)P2 in cytosolic calcium oscillation panels. *A,* the percentage of cells represent four type of cytosolic calcium oscillation patterns in Jurkat T-cells from 3 independent experiment. Cells were pre-incubated with increasing concentrations of exogenous PI(4,5)P2 before low concentration of anti-CD3 (1 μg ml^{-1}) stimulation. For each experiment, >30 cells of each group were quantified. *B,* the percentage of cells represent different number of calcium peak in cells with normal oscillation pattern. *P < 0.05, ***P < 0.001; Student's t test.
